# Measurements of Epidural Space Depth Using Preexisting CT Scans Correlate with Loss of Resistance Depth during Thoracic Epidural Catheter Placement

**DOI:** 10.1155/2015/545902

**Published:** 2015-01-01

**Authors:** Nathaniel H. Greene, Benjamin G. Cobb, Ken F. Linnau, Christopher D. Kent

**Affiliations:** ^1^Department of Anesthesiology and Pain Medicine, University of Washington School of Medicine, Seattle, WA 98195, USA; ^2^Department of Radiology, University of Washington School of Medicine, Seattle, WA 98195, USA

## Abstract

*Background.* Thoracic epidural catheters provide the best quality postoperative pain relief for major abdominal and thoracic surgical procedures, but placement is one of the most challenging procedures in the repertoire of an anesthesiologist. Most patients presenting for a procedure that would benefit from a thoracic epidural catheter have already had high resolution imaging that may be useful to assist placement of a catheter. *Methods.* This retrospective study used data from 168 patients to examine the association and predictive power of epidural-skin distance (ESD) on computed tomography (CT) to determine loss of resistance depth acquired during epidural placement. Additionally, the ability of anesthesiologists to measure this distance was compared to a radiologist, who specializes in spine imaging. *Results.* There was a strong association between CT measurement and loss of resistance depth (*P* < 0.0001); the presence of morbid obesity (BMI > 35) changed this relationship (*P* = 0.007). The ability of anesthesiologists to make CT measurements was similar to a gold standard radiologist (all individual ICCs > 0.9). *Conclusions.* Overall, this study supports the examination of a recent CT scan to aid in the placement of a thoracic epidural catheter. Making use of these scans may lead to faster epidural placements, fewer accidental dural punctures, and better epidural blockade.

## 1. Introduction

Local anesthetics and opioid medications administered by thoracic epidural catheters provide the best quality postoperative pain relief for major abdominal and thoracic surgical procedures [1]. The process of placing a catheter into the epidural space between the thoracic vertebrae can be challenging due to variations in thoracic spinal anatomy and narrow intervertebral spaces. One of the consequent risks is excessively deep placement of the needle with the potential complication of dural puncture headache and, rarely, needle induced injury to the spinal cord, making thoracic epidural placement a riskier procedure than a lumbar epidural.

Anesthesiologists increasingly use ultrasound imaging to guide the placement of needles into the body for the purpose of doing medical procedures. The bony vertebral column largely precludes the use of bedside sonography for steep angle neuraxial procedures owing to the lack of ultrasound transmission in bone. These challenges are magnified in the patients in whom neuraxial procedures are most difficult (those with morbid obesity or spinal abnormalities). While most anesthesiologists have a general idea at what depth they should expect to encounter the epidural space, there can be a wide range of variability between individuals [2]. The depth from the skin to the epidural space is influenced by both patient factors including body mass index [3] and procedural factors such as the needle angle required to enter the epidural space between the posterior elements of the vertebrae [4]. For any individual patient it would be helpful to know the specific depth to the epidural space prior to epidural needle placement.

With increased utilization of computed tomography (CT) for not only diagnostic purposes but also preoperative planning, most patients undergoing epidural catheter placement for pain control after major thoracic and abdominal surgeries will have recent abdominal and thoracic imaging available for review preoperatively.

The goal of this study was to compare measurements taken by anesthesiologists from CT imaging to the depth of loss of resistance as recorded during epidural placement. The secondary goal of this study was to compare the ability of anesthesiologists to measure this distance on a CT scan to a radiologist.

## 2. Materials and Methods

### 2.1. Patient Selection

**Figure 1:**
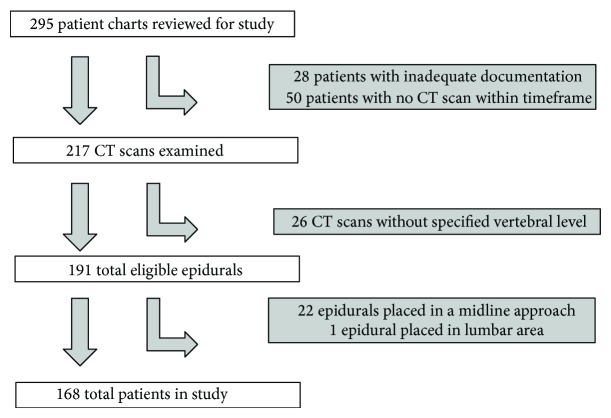
Patient selection procedure.

After local IRB approval, we conducted a retrospective cohort study using electronic medical records to identify eligible patients. Individual patient records were assessed for documentation of placement of a thoracic epidural catheter between March and August of 2011 at the University of Washington Medical Center. Patients were excluded if a CT scan of the respective spinal level was not available either 6 months prior to or during hospitalization following epidural catheter placement. If a patient had more than one appropriate imaging study, the scan closest in time to the epidural placement date was used. Given the high proportion of thoracic epidurals placed in the paramedian approach, we excluded thoracic epidurals placed in a midline approach from this analysis. We initially examined 295 patient records and included 168 records in our study (Figure 1).

### 2.2. Measuring Imaging Distances

**Figure 2:**
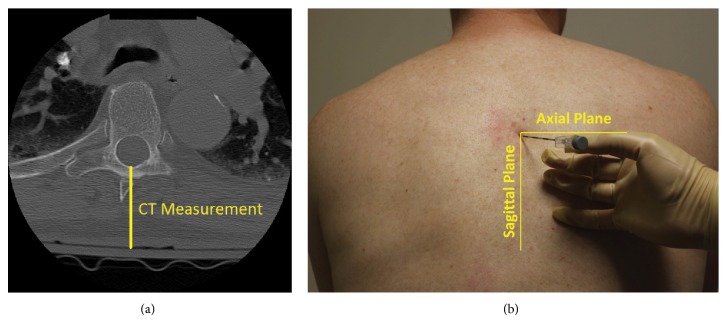
(a) Axial computed tomography (CT) image of thorax with demonstrated measurement technique; (b) demonstration of appropriate planes with epidural needle placed on back.

All images were viewed using the institutional online PACS (Centricity Enterprise Web; GE Healthcare, Waukesha, WI) tool with measurements being made within the program. After identification of the image that optimized the view of both pedicles of the upper vertebra of the interspace accessed when placing the epidural catheter (e.g., T7 vertebra if a T7-T8 epidural catheter was placed), a line was drawn between midline at the skin and the most posterior part of the spinal canal (Figure 2). This epidural-skin distance (ESD) was measured in the axial plane (Figure 2) and was recorded from the value automatically generated by the PACS software when drawing the connecting line. Measurements for all 168 epidurals made by the lead author of the study were used for analysis.

### 2.3. Assessing Anesthesiologists' Radiologic Abilities

In order to ensure an anesthesiologist would be able to reliably measure the distance from the skin to the epidural space, the imaging measurements made by three different anesthesiologists (Nathaniel H. Greene, Christopher D. Kent, and Benjamin G. Cobb) were compared with a radiologist who specializes in imaging of the spine (Ken F. Linnau). A random sample of 50 patients of the 168 in the study was selected for review by all authors. Each author independently made their own measurements of the epidural-skin distance. Agreement between the measurements made by anesthesiologists and the gold standard radiologist was assessed by calculating exact concordance, concordance within 5 and 10 mm, root mean squared error (RMSE), and intraclass correlation coefficient (ICC) for each anesthesiologist.

### 2.4. Clinical Prediction Rules

Based on simple trigonometric principles, we hypothesized that the ESD should be linearly related to the needle path when placing an epidural in a paramedian approach. The linear transformation required could theoretically be estimated using the angles of approach in the axial plane and sagittal plane (Figure 2). Thus, fitting a linear regression model using ordinary least squares would seem to best approximate the true relationship between the ESD and loss of resistance (LOR) depth. The best fit line would yield an equation with a slope and an intercept. Given that trigonometric principles demonstrate that our CT measurement should be directly proportional to the loss of resistance depth, there is likely some degree of tissue compression which would make us predict* a priori* that there will be a positive intercept value to account for this fact. It is likely that tissue compression would be higher in morbidly obese patients, as a greater proportion of the CT measurement is compressible adipose tissue. Because some of our patients were morbidly obese (BMI > 35), we included a variable in our model that reflected the presence of morbid obesity. Multivariable linear regression assuming robust standard errors was used with LOR depth as the dependent variable and ESD and presence of morbid obesity as an independent variable to test for association. As we hypothesized this CT measurement would be most useful to identify patients with abnormally shallow and abnormally deep epidural spaces, receiver operating curves (ROC) were generated for two outcomes, LOR depth less than or equal to 4 cm and LOR depth greater than or equal to 8 cm. All statistical analyses were done using Stata Intercooled 12 (StataCorp; College Station, TX).

## 3. Results

### 3.1. Patient Characteristics

There were a total of 166 patients analyzed (Table 1). They had an average age of 57 years and ranged from 20 to 90 years old. The average BMI was 28, with wide range represented from 15.5 to 53.9. Approximately 13% of the sample had a BMI of greater than 35. The population was 51% male and all portions of the thoracic spine were represented with epidurals being most commonly placed at the T8-T9 interspace (28%).

### 3.2. Assessing Anesthesiologists' Radiologic Abilities

There was significant agreement between all three anesthesiologists' and the radiologist's measurements with expected variance as evidenced by the lack of agreement when the measurements were the same (Table 2). Additionally, the root mean squared error was consistently low, with the maximum error of 3.7, approximately 8% of the mean measurement of 47 mm. All anesthesiologists' measurements also exhibited a very high intraclass correlation (>0.9) with the radiologist's measurement (gold standard).

### 3.3. Clinical Prediction Rule

**Figure 3:**
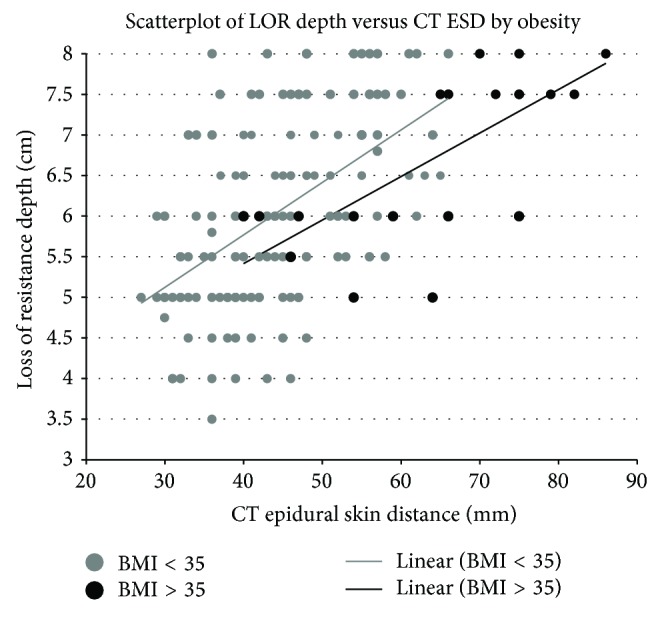
Scatterplot of loss of resistance (LOR) depth versus epidural-skin distance (ESD) separated by the presence of morbid obesity. Best fit linear lines projected on data.

**Figure 4:**
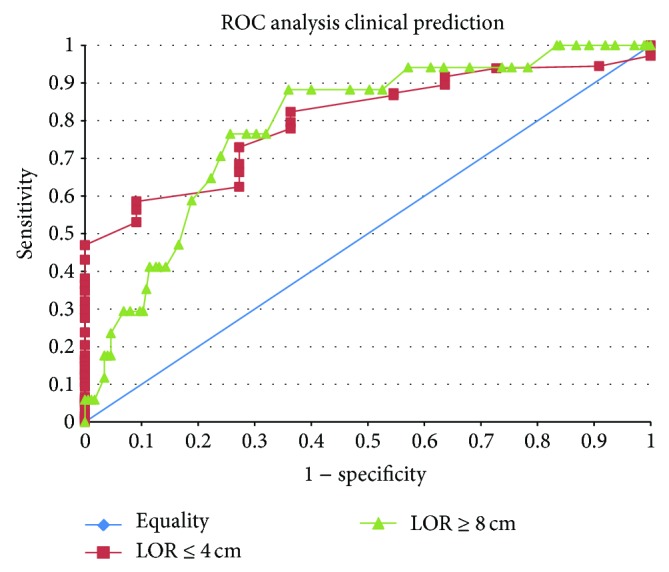
Receiver operating curve (ROC) of using epidural-skin distance (ESD) as measured by computed tomography (CT) to predict shallow or deep loss of resistance depths.

There appears to be a linear relationship between loss of resistance depth and epidural-skin distance (Figure 3). This relationship appears to be slightly different in the presence of obesity. Linear regression suggests a strong relationship between ESD and LOR for paramedian epidurals. Obesity was also significantly associated with loss of resistance depth after adjusting for the CT measurement. The intercept in the model was significantly above zero as well (Table 3). The area under the curve (AUC) of the ROC generated using ESD to predict a LOR greater than or equal to 8 cm was 0.7876 while the AUC of the ROC generated using ESD to predict a LOR less than or equal to 4 cm was 0.8009 (Figure 4).

## 4. Discussion

We found a strong relationship between the distance from the skin to the epidural space as measured on CT imaging and the clinically recorded depth of loss of resistance. The linear model appeared to be different for morbidly obese patients. The ESD measurement appeared to perform reasonably well in identifying very deep loss of resistance depths, potentially requiring the use of a nonstandard Tuohy needle, and very shallow depths, possibly alerting a clinician to prevent an inadvertent dural puncture. The anesthesiologists' measurements of this distance were very similar to the radiologist's measurements, indicating anesthesiologists may not need the assistance of a radiologist to measure the distance between the skin and the epidural space.

One limitation of our analysis is that it is retrospective in nature. There was no way to verify the loss of resistance depth each practitioner reported as the true loss of resistance. In our experience, clinicians may not always be completely accurate with the values reported, as documentation of placement of the epidural can be somewhat removed in time from the time of the procedure. Also, our analysis relies on the accurate identification of the vertebral interspace at which the epidural catheter was placed. It has been shown [5, 6] that clinicians are commonly one or two interspaces away from the targeted space. The relative precision of the CT ESD and LOR depth measurements are also dissimilar. The radiological software we used gave us precision down to the millimeter while the finest degree of precision routinely reported within loss of resistance measurements is down to 0.5 cm. When placing a thoracic epidural, it is also possible for a clinician to start at one interspace and traverse up to one or two interspaces if a steep angle is used thus obtaining a much deeper LOR than would have been produced by a more perpendicular approach. We also could not verify that the putative epidural catheter was in the true epidural space. Of the 168 epidurals examined, there were over 20 different anesthesiologists who actually performed the catheter placement. Based on our clinical experience with these anesthesiologists, there were several different techniques utilized which likely resulted in varying angles of approach which is the most likely explanation why there was still such variation in LOR depths for a given CT measurement distance.

Despite these limitations, this study shows the potential impact that careful review of already available CT scans can provide. Our findings are in agreement with two other published studies that have found good correlation between CT scan measurements to loss of resistance depth [4, 7]. The information derived from these previous studies is somewhat limited in its clinical utility. Both studies examine only one or two interspaces whereas epidurals can be placed at almost any thoracic level as required by the location of the surgical incision. In both of these previous studies, the description of how the measurements were made was not specific enough to indicate how an anesthesiologist might reproduce and use measurement information taken from a CT scan. This study helps support that the examination of a CT scan can be applicable at various interspaces in the thoracic spine. Additionally, the measurements can easily be made by anesthesiologist without the assistance of a radiologist. The anesthesiologists in this study have had no additional formal training in radiology outside of what was learned in medical school and anesthesiology residency.

This study also adds benefit in interpreting the CT measurements differently for obese patients. As can be seen in the scatterplot and best fit lines, although morbidly obese patients tended to have deeper LOR depths and higher ESD measurements, the relationship is different. As can be inferred from the linear regression model, the coefficient is negative suggesting shallower LOR depths than a normal weight patient with a similar CT measurement. This makes sense scientifically, as one would expect the tissue measured in a morbidly obese patient would be more compressible and an adjustment would need to be made. The true degree of compression seen in the CT image may also depend on the interspace as CTs are taken in the supine position and the kyphosis of the spine dictates differential pressures on the tissue posterior to the spine potentially causing differential compression.

Given the large number of patients examined in this study, the results do seem generalizable despite a relative lack of precision of our model.* A priori*, we predicted a pure linear transformation based on an angle in the sagittal plane of 30° and an angle in the axial plane of 15° which would estimate a ratio of 1.2. This line can be drawn on the scatterplot and approximate some of the data, but our ordinary least squares regression suggests that this is not the best fit model. The true model describing the relationship between CT ESD and LOR depth probably does have an intercept that accounts for tissue compression and a coefficient that is based on the angles of approach, but we cannot expect that a study of 168 epidurals in different patients would have the same angles of approach making the coefficient the aggregate “average” of coefficients calculated by the various angles used to place an epidural.

The value of having a sense of distance to the epidural space prior to starting a procedure could be particularly helpful. For example, if the predicted depth by imaging was never less than the actual loss of resistance depth then overly tentative epidural placements with false loss of resistance at shallow depths could be avoided. Closer examination of the scatterplot almost seems to reveal such a relationship in both populations, although this population is different. In our sample, 8% of patients had observed loss of resistance depths of 8 cm or greater suggesting that a longer nonstandard needle may have been useful. On the other end of the spectrum and perhaps more importantly, epidural-skin distance on imaging appears to also be useful in predicting abnormally shallow loss of resistance depths, which could help prevent dural punctures and thus nerve injury with potential for permanent damage.

## 5. Conclusions

In summary, our results suggest that making a simple measurement on a CT scan of a patient prior to placing an epidural catheter can be helpful in determining loss of resistance depth. This measurement can be adequately made by an anesthesiologist without the assistance of a radiologist. Further prospective data are needed to determine how precise this prediction could be and future studies should consider addressing obese populations separately.

## Figures and Tables

**Table 1:** Patient characteristics of sample (*N* = 166).

Continuous variable	Mean (SD)	Range
Age	56.8 (15.1)	20–90
BMI	28.1 (7.0)	15.5–53.9
CT epidural skin distance (mm)	46.7 (11.7)	27–86
Loss of resistance depth (cm)	6.1 (1.1)	3.5–8^*^

Categorical variable	Number	%

Male (%)	85	51
BMI > 35 (%)	21	13
Epidural level (%)		
T3	2	1.2
T4	9	5.4
T5	16	9.6
T6	24	14.5
T7	27	16.3
T8	46	27.7
T9	23	13.9
T10	12	7.2
T11	4	2.4
T12	3	1.9

^*^LOR depth > 8 classified as 8 (see text).

**Table 2:** Analysis result.

Anesthesiologists' measurements versus radiologist's measurement			
	Anesthesiologist 1	Anesthesiologist 2	Anesthesiologist 3
Measurements within 1 cm	98%	98%	100%
Measurements within 0.5 cm	98%	96%	86%
Measurements were the same	22%	34%	18%
Root mean squared error	2.5	2.3	3.7
Intraclass correlation coefficient	0.98	0.99	0.97

**Table 3:** Regression analysis.

Multivariable regression analysis			
	Coefficient	95% Cl	*P* value
CT epidural skin distance (mm)	0.053	[0.050, 0.075]	<0.0001
BMI > 35	−0.59	[−1.01, −0.16]	0.007
Constant	3.30	[2.71, 3.88]	<0.0001
